# Why Some Women Look Young for Their Age

**DOI:** 10.1371/journal.pone.0008021

**Published:** 2009-12-01

**Authors:** David A. Gunn, Helle Rexbye, Christopher E. M. Griffiths, Peter G. Murray, Amelia Fereday, Sharon D. Catt, Cyrena C. Tomlin, Barbara H. Strongitharm, Dave I. Perrett, Michael Catt, Andrew E. Mayes, Andrew G. Messenger, Martin R. Green, Frans van der Ouderaa, James W. Vaupel, Kaare Christensen

**Affiliations:** 1 Unilever Discover, Sharnbrook, Bedfordshire, United Kingdom; 2 The Danish Twin Registry and Danish Aging Research Center, Institute of Public Health, University of Southern Denmark, Odense, Denmark; 3 Dermatological Sciences, University of Manchester, Salford Royal Hospital, Manchester, United Kingdom; 4 Perception Lab, School of Psychology, University of St Andrews, St Andrews, United Kingdom; 5 Institute for Ageing and Health, Newcastle University, Newcastle Upon Tyne, United Kingdom; 6 Department of Dermatology, Royal Hallamshire Hospital, Sheffield, United Kingdom; 7 Netherlands Consortium for Healthy Ageing, Leiden University Medical Centre, Leiden, Netherlands; 8 Max Planck Institute for Demographic Research, Rostock, Germany; University of Exeter, United Kingdom

## Abstract

The desire of many to look young for their age has led to the establishment of a large cosmetics industry. However, the features of appearance that primarily determine how old women look for their age and whether genetic or environmental factors predominately influence such features are largely unknown. We studied the facial appearance of 102 pairs of female Danish twins aged 59 to 81 as well as 162 British females aged 45 to 75. Skin wrinkling, hair graying and lip height were significantly and independently associated with how old the women looked for their age. The appearance of facial sun-damage was also found to be significantly correlated to how old women look for their age and was primarily due to its commonality with the appearance of skin wrinkles. There was also considerable variation in the perceived age data that was unaccounted for. Composite facial images created from women who looked young or old for their age indicated that the structure of subcutaneous tissue was partly responsible. Heritability analyses of the appearance features revealed that perceived age, pigmented age spots, skin wrinkles and the appearance of sun-damage were influenced more or less equally by genetic and environmental factors. Hair graying, recession of hair from the forehead and lip height were influenced mainly by genetic factors whereas environmental factors influenced hair thinning. These findings indicate that women who look young for their age have large lips, avoid sun-exposure and possess genetic factors that protect against the development of gray hair and skin wrinkles. The findings also demonstrate that perceived age is a better biomarker of skin, hair and facial aging than chronological age.

## Introduction

The ability to estimate age has evolved as it enables an individual to evaluate, for example, the suitability of a potential mate [1], [2]. Perceived age is socially relevant to many individuals as evidenced by the large and global cosmetics industry. In addition, perceived age has been shown to be predictive of mortality in elderly individuals and associated with predictors of age-related diseases independently of chronological age [3], [4], [5] indicating its utility as a biomarker of aging. Biomarkers of aging are measures of an individual's or tissue's biological age (i.e. how well they are aging considering their chronological age) and are important in gerontology and epidemiology research for identifying factors that influence the aging process. However, there has been little work to systematically determine which physiological features predominately influence how old women look for their age and, hence, which features drive the link between perceived and biological age.

The influence of particular features on the perception of age depends on the context in which a subject is viewed. For example, photographs can be viewed with or without hair and clothing cues (e.g. passport-type [6] versus facial images [7]), and in-person evaluations of age [4] can be influenced by speech and body movement cues. The benefit of using photographic images is that the cues present for age estimation can be controlled and standardized. Additionally, estimating age from images has been shown to be highly reproducible when employing large numbers of age assessors [7]. Thus, photography is now the predominant method for generating perceived age.

Previous perceived age work has found that increased sun-damage [8], male pattern baldness [4], [9], gray hair [4], under eye wrinkles and bags [10], pigmented spots [10], skin topography (i.e. skin micro-texture and wrinkles) [11] and reduced skin color uniformity [10]–[12] are associated with looking older for one's age. However, the use of different methods to estimate perceived age and the different features measured in each study make it difficult to ascertain which skin and hair aging features dominate the perception of age. Additionally, it is unclear how the use of different photographic images in each study affects the relationship between perceived age and skin and hair aging features. Modern cosmetic surgical techniques target subcutaneous tissues to create a youthful look [13]–[16] suggesting that physiological changes underneath the skin are important modulators of how old one looks. Evidence for the link, though, between changes to subcutaneous tissue and age perception is limited, partly due to the difficulty in measuring subcutaneous changes.

The rate and degree to which physiological changes occur are determined by genetic and environmental factors. For example, around 80% of the variation in male pattern baldness in young and old men can be attributed to genetic factors [9], [17]. However, the degree of influence genetic factors have on features of female appearance are largely unknown. For example, whether it is sun-exposure or genetic factors that mainly underlie the variation in sun-damaged skin present in Caucasian populations is unknown. Estimating the influence genetic factors have on a particular feature can indicate, for example, the utility of using Genome Wide Association (GWA) approaches to investigate the etiology of the feature.

Here, to investigate the link between perceived age and biological age we have investigated the strength of relationship between how old women look for their age and skin, hair and lip aging features. Additionally, we investigated the impact on such relationships of using different types of photographic image to generate perceived age. To determine whether future investigations into the identity of factors that influence facial aging should focus on genetic or environmental factors, we have utilized monozygotic and dizygotic twins to estimate the influence genetic factors have on perceived age as well as on skin, hair and lip aging features. Furthermore, we have constructed composite images of individuals who look either young or old for their age to demonstrate the striking differences in perceived age that can occur between individuals of the same chronological age.

## Results and Discussion

### 2.1 Features Associated with Perceived Age

**Figure 1:**
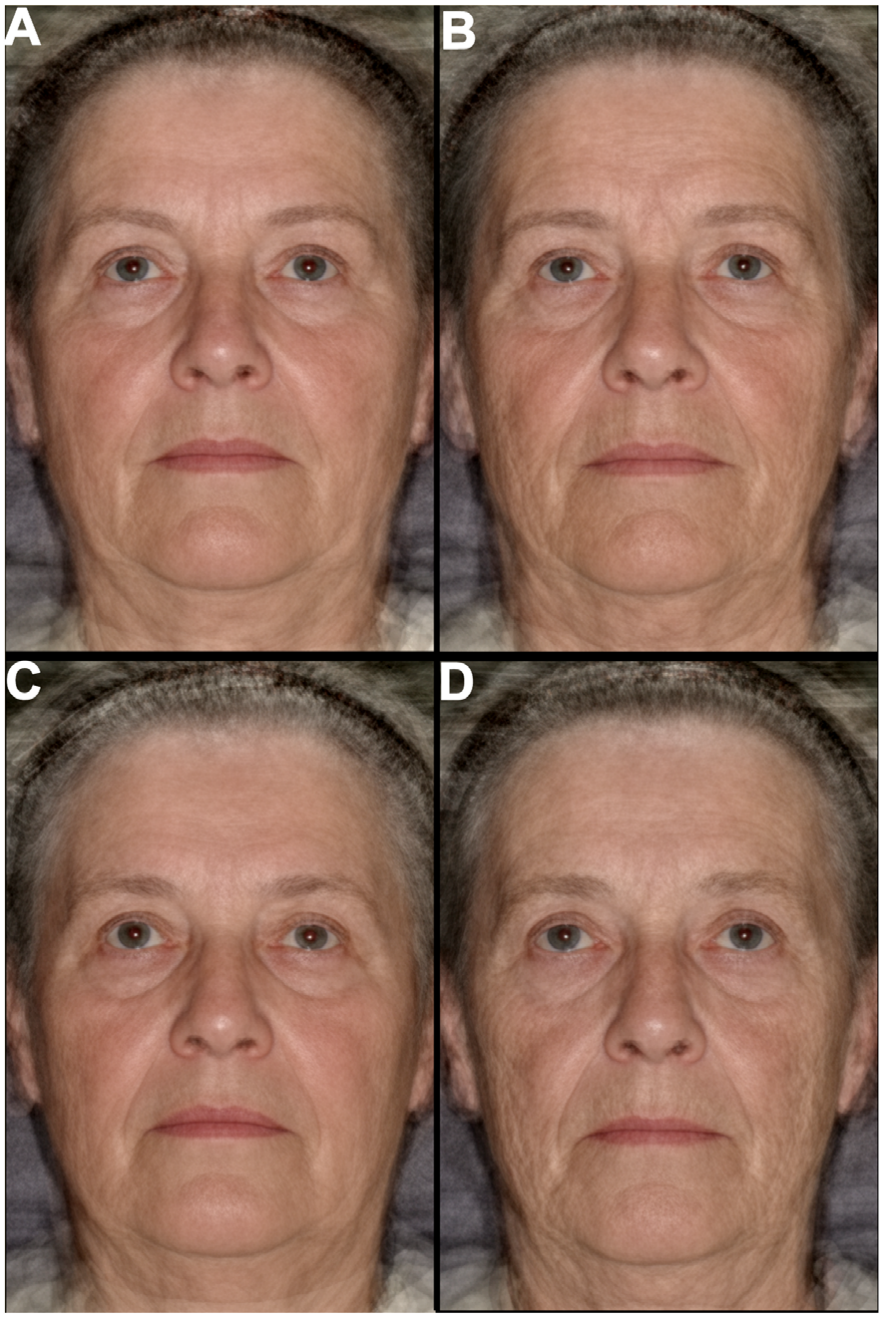
Composite images representing the effects of environmental factors on variation in perceived age between monozygotic twin sisters (upper images) and the effects of environmental and genetic factors on variation in perceived age between dizygotic twin sisters (lower images). **a**, Younger looking and **b**, older looking monozygotic twin sister composites (mean perceived age 64 [57–70] and 70 [60–85] respectively). **c**, Younger looking and **d**, older looking dizygotic twin sister composites (mean perceived age 64 [59–74] and 76 [69–84]). The older looking twin sister composites demonstrate signs of increased skin wrinkling, increased nasolabial fold shadowing (running from the lateral edge of the nose to the outer edge of the mouth) and, particularly for the non-identical twin comparison, a grayer skin color, a thinner face and reduced lip fullness. Each composite image was derived from 14 twin images and the chronological age was 67 [60–76] and 69 [61–79] for the monozygotic and dizygotic composites respectively; square brackets denote age ranges.

Monozygotic and dizygotic Danish female twins aged 59–81 years had their age assessed, by an average of 71 assessors, in facial photographic images that had been cropped around the lower neck and scalp hair line (see Methods section 3.2 for further details). The resulting mean estimates of perceived age revealed that some twin sisters looked considerably different in age from each other. To examine why such differences existed, four composite (or average) facial images were created from the 14 most discordant perceived age monozygotic and dizygotic twin pairs (Fig. 1). Composite images are created by merging together facial images using the facial shape, skin color and skin topology information present in each image. Such composite images have been shown to reflect the ages of the individuals used to create the images [12], [18] and, thus, capture some of the changes in facial appearance that occur with age. There were marked differences between the Danish twin composite images in the appearance of skin color and topology as well for features of face shape, particularly the size of the lips and the nasolabial fold (Fig. 1). The differences between the dizygotic twin composites were more striking than the monozygotic twin composites indicating an influence of genetic factors on perceived age.

**Figure 2:**
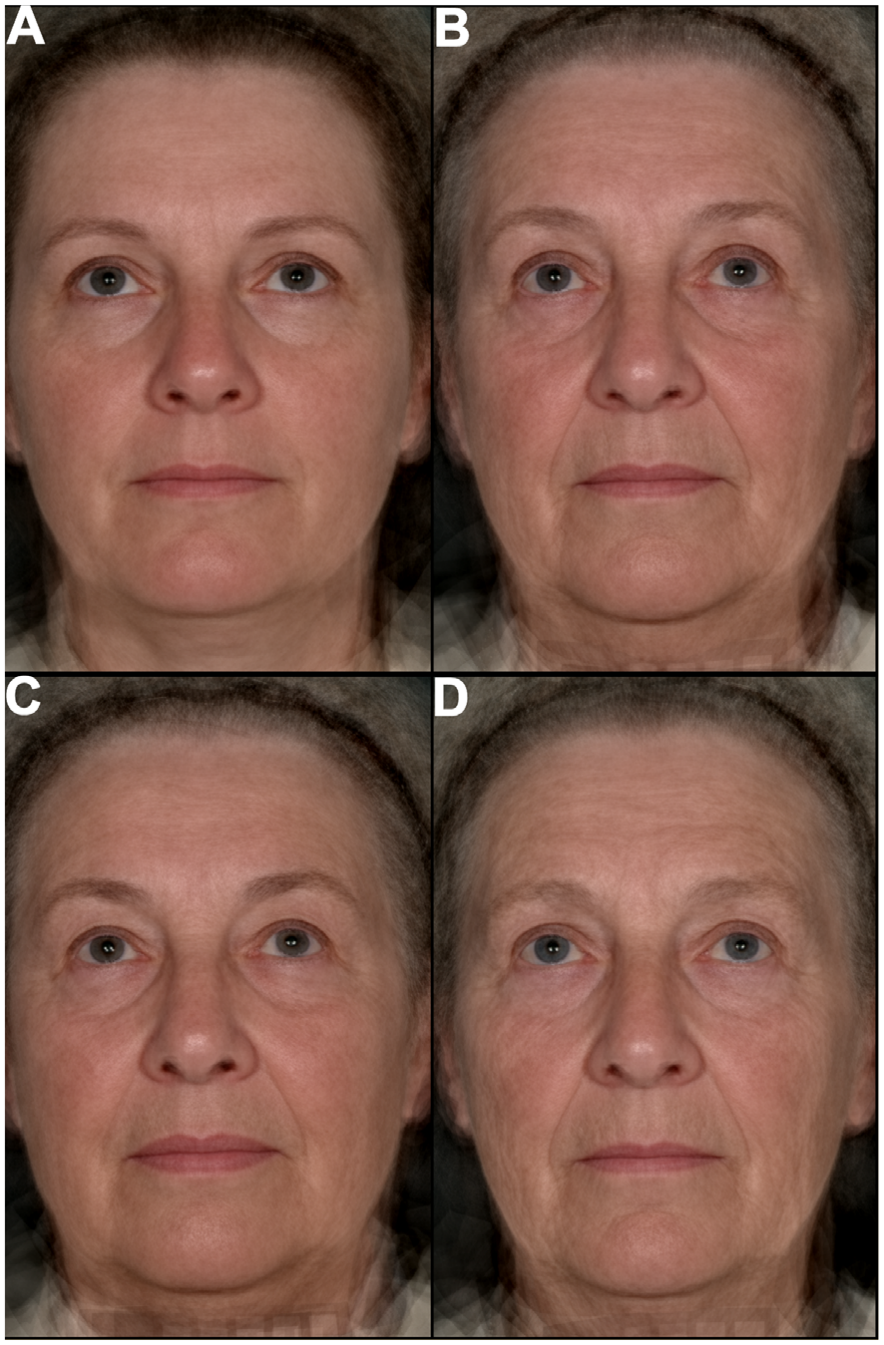
Composite images representing the average differences between 50 and 70 year olds (upper images) and 70 year old young and old looking subjects (lower images) in the British population. Composite images of **a**, 50 year olds (mean chronological age 50 [48–52]) and **b**, 70 year olds (mean chronological age 70 [68–72]). **c**, Young looking 70 year olds (mean perceived age 62 [57–68]) and **d**, old looking 70 year olds (mean perceived age 73 [67–79]) composite images. Differences in skin wrinkling and the nasolabial fold (upper and lower images) and lip fullness (lower image) are similar to those in the twin composites (Figure 1). The upper images were each derived from 18 female images and the lower images from 17. The mean chronological age was 71 [67–75] and 70 [66–74] for the young and old looking 70 year old composites respectively; square brackets denote age ranges.

To confirm that the visual differences in evidence between the Danish twin composites were reflective of facial aging differences in other Caucasian populations, perceived ages of British females aged 45–75 years were estimated. The estimated means were used to create 2 composite images representing women who looked either old or young for their age. In addition, 50 and 70 year chronological aged composites were created. Similar differences to those in evidence in the twin composite image comparisons were in evidence in the British perceived and chronological age composite comparisons (Fig. 2), confirming that the differences between the Danish twin composites were reflective of features that correlate with perceived age in Caucasian populations and change with age.

### 2.2 Skin Aging and Perceived Age

To determine to what degree perceived age is an indicator of skin aging in the face, skin wrinkling and pigmented age spot grading of the facial photographs and topographical analysis of skin beside the left eye (i.e. crows feet region) were carried out in the Danish twin and British populations. Perceived age data generated from the facial images were found to be significantly and strongly correlated, after controlling for chronological age, with facial skin wrinkling and wrinkle depth (a topography measure, see Methods 3.2) in the crows feet region in both populations (Table 1 and also see Supporting Information [SI] Fig. S1). Thus, skin wrinkling was strongly related to looking older for one's age confirming the visual differences present in the composite images (Figs. 1 and 2).

**Table 1:** Aging appearance feature inter-correlations after adjusting for chronological age.

Twin study					British study				
	Pigmented			Pigmented	**0.51** ***	**0.27** ***	0.03	−0.13	0.13
Sun-damage	**0.30** ***	Sun-damage			Sun-damage	**0.84** ***	**0.32** ***	**−0.28** ***	**0.50** ***
Wrinkles	**0.16** *	**0.92** ***	Wrinkles			Wrinkles	**0.33** ***	**−0.34** ***	**0.53** ***
Wrinkle depth	0.00	**0.43** ***	**0.46** ***	Wrinkle			Wrinkle depth	**−0.17** *	**0.36** ***
Lip height	0.03	−0.04	−0.03	−0.02	Lip height			Lip height	**−0.47** ***
Perceived age†	0.10	**0.69** ***	**0.74** ***	**0.38** ***	**−0.17** *	Perceived age†			Perceived age†
Perceived age‡	0.01	**0.26** ***	**0.29** ***	**0.18** **	−0.13	**0.55** ***	Perceived age‡		
Hair graying	0.02	−0.12	−0.12	−0.06	−0.06	−0.14	**0.25** **	Hair graying	
Hair recession	0.10	−0.01	0.02	0.05	−0.01	0.12	0.16	0.04	Hair recession
Hair thinning	0.05	0.01	0.02	0.14	−0.07	0.01	**0.17** *	**0.29** **	**0.21** **

* <0.05.

** <0.01.

*** <0.001.

† facial image derived.

‡ passport-type image derived.

The appearance of skin wrinkling is one of the main features indicative of the severity of sun-damage present in faces, predominantly when it appears alongside other features typical of sun-damage in exposed body sites [19]. To determine the strength of relationship between sun-damage and perceived age, grading of the images for the presence of features consistent with sun-damage was carried out. Sun-damage was significantly and strongly correlated to the perceived age data generated from facial images in both the Danish twin and British populations (Table 1) supporting evidence that sun-exposure is associated with looking older for one's age [6]. In addition, both facial skin wrinkling and wrinkle depth in the crows feet area were significantly and strongly correlated to sun-damage in both populations after controlling for chronological age, similar to findings reported in the literature [8], [19], [20]. Hence, chronic sun-exposure was likely to have caused some of the differences in skin wrinkling apparent between the younger and older looking composites (Figs. 1 and 2). However, sun-exposure is not the only factor that influences the development of skin wrinkles. For example, repeated skin contouring caused by forces external to the skin [21], [22] and by muscle contractions [23] have also been implicated in the development of skin wrinkles.

There was a significant correlation between the perceived ages of the British subjects and the pigmented spot grading without adjusting for chronological age (see SI Table S1), similar to findings in the literature [24]. However, pigmented age spots did not significantly correlate with perceived age in either population after adjusting for chronological age (Table 1), in contrast to a previously reported study [10]. Further work is required to determine whether study power, methodologies or the subjects examined underlie the difference between the findings. Here, although related to perceived age per se, pigmented age spots were not significantly correlated to how old women look for their age.

The pigmented spot grading had a significant but weak to moderate correlation with wrinkles and sun-damage after adjusting for chronological age (Table 1). These data highlight the complexity of the relationship between pigmented spots, skin type and sun-damage; for example, actinic lentigines are more prevalent in Asian than Caucasian skin [25] whereas freckles are more prevalent in fair-skinned than dark-skinned Caucasians [26]. Hence, although sun-exposure causes an increase in the number of pigmented spots, it does so differentially depending on the skin type of the individual, which also determines whether they present alongside other signs of sun-damage such as skin wrinkling.

### 2.3 Hair Aging and Perceived Age

No scalp hair cues were present in the cropped facial images used to generate perceived age (see section 3.2). However, hair aging features could be important modulators of age perception. Therefore, hair recession (hair loss on the frontal and temporal region of the head), hair thinning on the top of the head (female pattern hair loss) and hair graying were measured in the Danish twin population along with how old the twins looked in passport-type photographs. Hair thinning and graying were found to be weakly but significantly correlated with each other (Table 1), although this link could be partly due to the measurement technique (SI Discussion). Hair graying and hair thinning but not hair recession were found to be significantly correlated with how old the Danish twins looked for their age in the passport-type images (Table 1 and also SI Fig. S1). Hair recession was not in evidence in some of the passport-type images due to subjects' hair-styles. This could account for the lack of any significant correlation between hair recession and perceived age (Table 1). There was also no significant correlation between the hair features and perceived age when hair cues were removed from the images (i.e. the facial images of the Danish twins). This indicated there could be a positive causal relationship for hair graying and thinning with perceived age. Data were collected from the Danish twin participants on the use of hair colorants. Those who reported that they used hair colorants looked significantly younger for their age than those who did not in the passport-type images (differences in mean perceived ages when controlling for chronological age was 1.69 years, F-test p-value = 0.0045) but not in the facial images. Therefore, the link between perceived age and gray hair was causal in nature rather than associative.

A comparison between the perceived ages of the Danish twins in the facial images and the passport-type images, generated by the same assessors, revealed that the twins looked older for their age in the facial images (differences in perceived age means was 4.16 years, F-test p-value<0.001). Taken in conjunction with the hair colorant result, the cues present in the passport–type images that can be modified to convey youthfulness (i.e. via the use of hair colorants, make-up, clothing and jewelry) were most likely responsible for the twins looking younger in the passport-type images than in the facial images. There was no significant correlation for any of the hair aging features with the skin aging features or the perceived age data generated from the facial images. However, despite the differences between the facial and passport-type images the correlation between the perceived age data from the facial images and the passport-type images was high (Table 1). Thus, twins who looked young for their age generally did so in both types of image.

### 2.4 Perceived Age as a Marker of Biological Age

It is important in gerontology research to utilize markers of the biological age of an individual and/or their tissues to enable the identification of factors that influence aging over and above generational differences that chronological age (or a proxy thereof) would identify in a population. Here, perceived age was found to be a good biomarker of aging as significant correlations were found between skin and hair aging features and perceived age after adjusting for chronological age (Table 1). Differences in lip size were in evidence in the composite comparisons (Figs. 1 and 2). To verify that lip size was linked to how old women looked for their age, lip height was measured (as an indicator of lip size) in the photographs of both the Danish twin and British populations and was also found to be significantly correlated with perceived age in the facial images of both populations after adjusting for chronological age (Table 1). Therefore, perceived age was a good marker of the biological age of the skin, hair and lips and has utility in gerontology studies over and above the use of chronological age.

To determine whether the skin, hair and lip features were related to perceived age independently of each other as well as chronological age, linear regression models were utilized to predict perceived age in both populations. For the perceived ages generated from the Danish twin passport-type images and excluding those who used hair dye, only skin wrinkles and hair graying had significant prediction in a linear model (Table 2). When hair graying was excluded from the model, hair thinning became a significant predictor of how old women look for their age (SI Table S2). This suggests that hair thinning might only have correlated to how old women looked for their age because it was a proxy of hair graying. Male pattern baldness makes young but not elderly men look older for their age [9]. Hair thinning might, therefore, have a greater influence on how old women look for their age in younger age groups.

**Table 2:** Multivariate linear modeling to predict perceived age.

Feature	Perceived age Danish twins (n = 204)	Perceived age Danish twins passport-type images (n = 158)	Perceived age British subjects (n = 162)
Chronological age	0.51 (0.05)***	0.53 (0.06)***	0.72 (0.04)***
Pigmented spots	N/S	N/S	N/S
Sun-damage	N/S	N/S	N/S
Wrinkles	2.92 (0.18)***	0.95 (0.23)***	1.75 (0.27)***
Lip height	−0.55 (0.17)**	N/S	−0.83 (0.17)***
Hair graying	N/S§	2.0 (0.57)***	N/A
Hair recession	0.92 (0.39)*	N/S	N/A
Hair thinning	N/S	N/S	N/A

The slope, the standard error of the slope (in brackets), and the statistical significance of each feature in the models are given.

* <0.05.

** <0.01.

*** <0.001, N/S–not significant in model, N/A–not available.

§ - see Methods 3.4.

For the Danish facial images, wrinkles, lip height and hair recession were independently predictive of how old the twins looked for their age (Table 2). As the facial images were cropped below the hair line, hair recession was either influencing age perception via the size of the forehead visible in the images or was correlated to (i.e. a proxy for) an unknown facial feature in the image. For the British subjects, skin wrinkling and lip height independently predicted how old women looked for their age supporting the correlation findings in Table 1. However, the appearance of sun-damage had no significant independent prediction of how old women looked for their age in any of the models, but was predictive if skin wrinkling was excluded (SI Table S2). Thus, skin wrinkling was the main feature of sun-damage that influenced how old the women looked for their age.

The amount of variation in the perceived age data explained by chronological age and the aging appearance features included in the linear models were 73% and 86% for the facial images of the Danish twin and British subjects respectively; there was, therefore, considerable variation in the data unaccounted for. Age-related changes to subcutaneous tissue have been extensively documented and implicated as affecting facial appearance [13]–[16], and probably accounted for some of the remaining variation in the data. In support of this, the nasolabial fold has been linked to perceived age [10], was visibly different in the composite comparisons (Figs. 1 and 2), and changes to its appearance have been linked to changes in the redistribution of facial fat with age [27], [28]. Thus, further work to identify all the main features driving how old one looks for one's age will help determine what physiological features are primarily responsible for driving the link between perceived age and health [4], [5] and mortality [3].

### 2.5 Similarities in Aging Appearance between Twin Sisters

We assessed the similarity of features of facial appearance in mono- and dizygotic twins. The classic twin-study methodology is based on the fact that monozygotic twins have identical genotypes whereas dizygotic twins share, on average, half of their gene variants and, thus, are no more genetically related than ordinary siblings. A greater phenotypic similarity in monozygotic than dizygotic twin sisters is to be expected if there is a substantial genetic component in the etiology of the condition. By comparing the correlation in the appearance of a feature in monozygotic twin pairs to dizygotic twin pairs (see SI Fig. S1) it can be estimated how much of the variance in the feature can be attributed to genetic factors (i.e. its heritability), shared environmental factors (between twin sisters) and unique environment.

**Figure 3:**
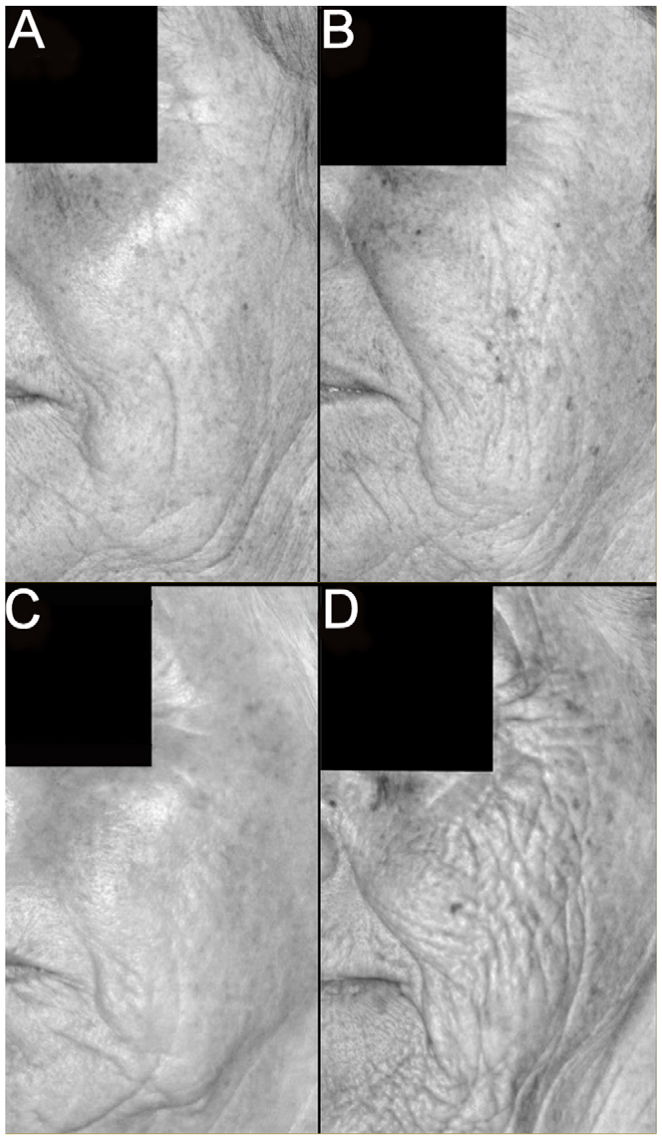
Example of the most discordant monozygotic twin pair (68 years of age, perceived facial age a 63 years and b 68 years) and dizygotic twin pair (71 years of age, perceived facial age c 71 years and d 82 years) for skin wrinkling grading. These images illustrate the greater differences in skin wrinkling that was found between dizygotic twins compared to monozygotic twins. Images are derived from the blue light channel of the photographs.

Heritability analyses of the skin aging features in the twin population demonstrated that 41–60% of the variation in sun-damage, skin wrinkling, wrinkle depth and pigmented age spot measures were explained by genetic factors (Table 3 and for an example of differences in twins see Fig. 3). In support of the wrinkle depth finding, 62% of the variation in skin topology profiles on the dorsum of the hand in Caucasians has been attributable to genetic factors [29]. The sun-damage heritability estimate indicated that genetic factors present in Caucasian populations were as important in the prevalence of sun-damage as sun-exposure. Gene variants that influence skin pigmentation in Caucasians are known [30]–[32] but it is not clear to what extent these variants also influence the prevalence of sun-damage. Both sets of perceived age data were found to be more or less equally influenced by environmental and genetic factors (Table 3), similar to findings from a different Danish twin study reported in the literature [3]. This finding supports the more marked visual differences in evidence in the dizygotic composite comparison than that found for the monozygotic composite comparison (Fig. 1). Although the identities of the genetic factors that influence perceived age are unknown, a number of environmental factors and conditions including social class, marital status and depression have been found to associate with perceived age in women [6].

**Table 3:** Heritability scores and technical reproducibility for the aging appearance feature measures.

Feature	Number of twins	Reproducibility	Monozygotic correlations††	Dizygotic correlations††	Best fitting model	Heritability (%)
Pigmented Spots	204	0.94§	0.44	0.15	AE	41 [14, 61]
Sun-damage	204	0.90§	0.60	0.06	ADE	60 [40, 73]
Wrinkles	204	0.95§	0.58	0.09	AE	55 [34, 70]
Wrinkle Depth	186	0.98¶	0.60	0.16	AE	57 [35, 73]
Lip Height	196	0.99¶	0.67	0.25	AE	66 [48, 78]
Perceived Age*	204	0.99‡	0.61	0.11	ADE	61 [41, 74]
Perceived Age†	226	0.92‡	0.51	0.22	AE	51 [32, 66]
Hair Graying	124	0.95**	0.90	0.34	AE	90 [80, 94]
Hair Recession	204	0.69§	0.81	−0.03	ADE	80 [51, 94]
Hair Thinning	204	0.61§	0.06	0.31	E	0 [0, 49]

* –facial image derived.

† –passport-type image derived.

‡ - Chronbach Alpha Test.

§ - Kendall's coefficient of concordance.

¶ –Pearson correlation coefficient.

** - Spearmann correlation coefficient.

†† - Intra-class correlations bar the hair recession and thinning which are tetrachoric, A–additive genetic effects, D–dominance genetic effects, E–unique environment, [ ] - 95% confidence intervals.

Here, we found that lip height was mainly influenced by genetic factors (Table 3). Lip size is established during development and then decreases in size during adult life-span [33]. Genes influencing lip size could be acting during either of these two periods or during both. Lip height is significantly correlated to perceived age in both populations and to skin wrinkling in the British population (Table 1). Estrogen levels have been linked to perceived age [34], skin wrinkling [35] and facial attractiveness [36], and lip size has been associated with female attractiveness [37]. Hence, it is plausible that some of the gene variants that influence lip height are doing so through their effects on estrogen biology.

Some genes have been implicated in the biological pathways that influence hair graying in mice [38] but genetic variation that influences the prevalence of human graying has yet to be identified. Here, we found a very strong influence of genetic factors on variation in hair graying (Table 3), similar to findings from a small twin study [39]. This indicates that GWA analysis of hair graying would be a feasible way to study the etiology of hair graying. For female hair recession, a similar influence of genetic factors was found (Table 3) to that reported for male pattern baldness [9], [17]. Therefore, along with a familial link between the features [40], hair recession in females and male pattern baldness are both mainly influenced by genetic factors. Hair thinning, in contrast, was negligibly influenced by genetic factors and was significantly but weakly correlated with hair recession. Hence, despite both hair recession and hair thinning being thought to result from the miniaturization of hair follicles [41], [42], the two features do not necessarily present together in individuals. Evidence for which environmental factors affect female hair loss is limited and conflicting (e.g. psychological stress [43], [44]) and further research is required.

### Summary

The size of influence that genetic factors can have on a feature tends to be dependent upon the environmental conditions in which a population exists [45]. Thus, limitations of the studies presented here include whether the heritability estimates are typical for Caucasian populations and other age ranges, the assumptions made to estimate heritability [45] and technical error. However, the technical reproducibility was fair to good dependent on the feature scored (Table 3 and SI Methods). Due to the large variation in skin and hair types between ethnic populations [25], replicate studies will be required to determine if these findings are similar in other ethnic groups.

Here, we have found that skin wrinkles, lip height and hair graying were significantly and independently correlated with how old women looked for their age. In addition, evidence that sun-exposure significantly influences how old women look for their age through its effects on skin wrinkling was found. Furthermore, we have quantified the influence of genetic factors on skin, hair and facial aging features and found evidence that subcutaneous tissue plays a role in how old women look for their age. Collectively, these findings will help direct future investigations into why appearances change with age and enable the use of perceived age, with consideration to the type of photographic image, in epidemiological approaches to identify the genetic and environmental factors that influence skin, hair and facial aging.

## Methods

### 3.1 Study Design

Caucasian twins aged 59–81 years were recruited in Denmark and informed written consent obtained. The study protocol was approved by the Research Ethics Committee for the Region of Southern Denmark. Zygosity established by the Applied Biosystems AmpFλSTR Identifiler kit indicated self-reported zygosity was accurate for all but 3 dizygotic twins; these twins were re-classified as monozygotic. British Caucasian subjects aged 45–75 years were recruited in southern England and gave informed written consent, and the study was approved by the Unilever Colworth Ethical Committee. Recruitment of the British and Danish subjects was carried out to give an even spread of subjects across the chronological age range of each study (SI Methods for further details and inclusion/exclusion criteria).

### 3.2 Feature Measures

Facial and passport-type photographs for 226 twins in Denmark and facial photographs of 185 women in Britain were acquired (as detailed elsewhere [7]). For the facial images, the subjects were free of make-up and the images were cropped around the neck and hair line (to remove clothing and scalp hair cues). A front-on view of the face alongside a 45 degree (°) angled view of the left side of the face were presented in a randomized order (to prevent biases from preceding images) to age assessors via a computer screen (see [7] for further details). The mean perceived ages were generated from an average of 71 and 51 independent assessments of age for 204 twins (52 monozygotic and 50 dizygotic pairs) and 162 British subjects respectively. Of the 53 age assessors who took part in the British study, 32 were part of the 102 assessors who assessed the age of the twin facial images. Assessors were predominantly British and Caucasian, and employees based at Unilever Research and Development sites; assessors were recruited via their response to advertisements. Although age assessors were of mixed gender and of varying age, assessor gender and age have been shown to have little effect on the mean perceived ages of subjects when large numbers of age assessors are used [7]. All 226 twins also had their age assessed in passport-type images (i.e. a front-on image of a subject from the chest upwards, including hair and make-up cues), presented in a randomized order via a computer screen, by 11 Danish age assessors (see [3], [6], [7] for further details). To enable comparison between perceived ages from facial and passport-type images, 10 of the same assessors also rated the twin facial images. The vertexes of the British subjects were not visible in the photographs restricting this population to measures of skin and facial aging.

For sun-damage, wrinkling and pigmented spot grading, an eyes closed front-on facial image was presented to a dermatologist side by side with a blue channel version of the same image to enhance the appearance of wrinkles and pigmented spots. Twin sister assessments were made either side of the British subject assessments and were separated by approximately 4 months to minimize any bias in the scoring of the twin assessed second. Grading was carried out for all three measures on a 9-point scale; the appearance of features consistent with sun-damage was graded according to a previously reported methodology and scale [46] and pigmented age spots and wrinkles as detailed in SI Methods.

In both populations, a skin replica was taken laterally to the left eye in the crows feet region of the face and its topography analyzed. The PRIMOS software Wt parameter generated from the skin replicas/molds is a measure of wrinkle depth [47] and had the highest correlation with perceived age (when chronological age was controlled for) and was used for further analysis.

A front-on and 45° photograph were used to score hair recession in the frontal temporal region using the Hamilton-Norwood scale (SI Fig. S2 and [17]) and, via the mirror placed above the subject's head, hair thinning on the crown using the Sinclair scale (SI Fig. S2 and [48]). Twin pairs were separated into two assessment sessions, with the second session being assessed at least one month after the first to minimize possible bias in the scoring of the twin seen second. Three assessors rated the images independently of each other and the median value was used for subsequent analysis. For hair graying, a 45° image was used to crop hair from the temporal area of the left-side of the head to create a new image and each image was then analyzed by a computer model to determine the percentage of pixels that were gray in color (see SI Methods and SI Fig. S2). The use of hair colorants in the twin study was ascertained via a questionnaire which first asked if subjects used hair colorants and then asked how long ago they had last used hair colorants (see SI Methods for further details).

For both populations, lip height was measured from the 45° photograph of the left-side of the subject's face by cropping the image from the highest point of the left-hand side of the lip (i.e. the vermillion border on the philtral crest) to the lowest point of the lips directly below, and another cropped image created from the top of the fore-head to the bottom of the chin (to measure face height). Variations in the lip height measures could have been caused by differences in the distance of the subject to the camera and by subject head size. Thus, the vertical height of the lip image was then measured in pixels and adjusted for face height in pixels (lip height/face height) to limit the impact of such variations and to benchmark lip height relative to the size of the face.

### 3.3 Generation of Composite Images

To generate the composite images, software was employed which merges together facial images by blending together the face shape, skin color and skin topology from each image. To capture face shape, one hundred and fifty six landmark points were located on each frontal eyes-open photograph creating a delineated image. The face shape position data was used to compute an average face shape, and the color information warped onto this average shape before the mean color values were calculated [12]. Finally, wavelet based techniques were employed to transform the appearance of skin topology from each image onto the composite; this prevents the smoothing of skin topology features whilst creating composite images [18].

### 3.4 Statistical Analysis

In the Danish twin study, for all analyses (bar the hair graying analysis and the assessment of age in the passport-type images) 22 of the twins were excluded due to either twin pair having non-cosmetic surgical treatments performed on their face and to balance the design of the assessment of age in facial images (see [7]). Due to technical failures, an additional 5 subjects were excluded from the lip height and 11 from the wrinkle depth measures (leaving 98 and 93 intact twin pairs respectively). A total of 68 subjects were excluded from the hair graying analyses mainly due to hair colorant use (see SI Methods). For the British study, 23 subjects were excluded from having their age assessed either because the subjects might have been known to some of the age assessors or to balance the design of the assessment of age [7].

All assessors were unaware of presentation designs, the presence of technical replicates, subject ages and age ranges. Technical reproducibility (Table 3) was examined for all the measures and is detailed further in Supporting Information S1. The feature data was corrected for chronological age by carrying out a linear regression of the data with chronological age and using the resultant residuals in the inter-feature Pearson correlation analysis. In order to overcome an underestimation of the variance due to data from within a Danish twin pair being highly correlated, the twin pairs were treated as clusters in the estimation of the correlations and their variances. A linear regression model was fitted to predict perceived age using a stepwise technique where the feature variables were entered into the model if they reached a significance level of <0.1, and variables were retained in the model if they achieved a significance level of <0.05. For the twin facial images, hair graying was not a significant predictor of perceived age when excluding those who used hair dye (data not shown); hence, to increase the power in the model, data is given for a model including those who had used hair dye which excludes the hair graying data (Table 3).

Differences in the feature data within twin pairs were compared between the monozygotic and dizygotic twins using intra-class correlations. For hair thinning and hair recession, as the majority of scoring occurred in the first two grades, the data were dichotomized for the heritability estimation and tetrachoric rather than intra-class correlations were calculated. Intra-pair correlations, tetrachoric correlations and heritability estimates were calculated by constraining the means and variances to be equal across the arbitrary categories of twin 1 and 2 and across zygosity. Structural equations were fitted (using Mx version 1.7.01) to a set of models of the twin data with all possible combinations of additive genetic effects, dominance genetic effects, shared environmental effects and unique environmental effects [49]. The heritability estimates were calculated as the sum of the additive and dominance genetic effects in the best fitting model selected on the basis of lowest Akaike information criteria.

## Supporting Information

Supporting Information S1 (0.04 MB DOC)

Figure S1 Scatter plots representative of correlations presented in the main manuscript. a) Facial wrinkling versus perceived age from facial images after adjusting for chronological age. b) Hair grayness versus perceived age from passport-type images after adjusting for chronological age. c) Wrinkle depth measures from beside the left eye for monozygotic twins (x-axis) and their sisters (y-axis). d) Wrinkle depth measures from beside the left eye for dizygotic twins (x-axis) and their sisters (y-axis). Graph lines are linear fits of the y-axis data onto the x-axis data and are representative of the correlation values in the main manuscript.
(6.20 MB TIF)

Figure S2 Representative images for hair aging measures. a) A 45-degree angle image of a subject with little to no hair recession (left-hand image) and a subject with grade 3 recession on the Hamilton-Norwood scale (right-hand side). b) An image of a subject with little to no hair thinning (left-hand image) and a subject with grade 3 thinning on the Sinclair scale (right-hand side). c) Example of a 45-degree image (left hand image) used to extract an area of hair from the left-hand temporal area of the head (centre image) and image analysis of gray (white) and non-gray (green) pixels in the image (right-hand image).
(4.43 MB TIF)

Table S1 Aging appearance feature and chronological age unadjusted inter-correlation values.
(0.05 MB DOC)

Table S2 Multivariate linear modeling to predict perceived age excluding hair graying or facial wrinkle measures.
(0.03 MB DOC)
