# Supplementary material for: Why Some Women Look Young for Their Age
Source: PLoS One. 2009 Dec 1;4(12):e8021. doi: 10.1371/journal.pone.0008021 (PMC2779449; doi:10.1371/journal.pone.0008021)
Supplement: Supporting Information S1 — (0.04 MB DOC) [file pone.0008021.s001.doc]

**Supporting Information**

**Discussion**

**Correlation between Hair Thinning and Graying**

The Sinclair scale was used for the measurement of hair thinning and utilizes photographic images of the vertex of the head with the scalp hair parted along the mid-line (main manuscript Methods and Fig. S2). The amount of scalp visible gives an indication of the amount of hair thinning and has previously been shown to be mainly caused by hair follicle miniaturization and then loss [50]. However, gray hair is more similar in color to the skin on the scalp of Caucasians than, for example, brown hair. Therefore, the correlation between hair thinning and graying hair could be due, in part, to the presence of gray hair giving the impression that there was greater visibility of the scalp than there actually was in the photographs. However, a biological link between hair graying and hair thinning could also have been driving the positive correlation.

**Methods**

For both studies, subjects were recruited by sending information packs to those on specific databases (Danish Twins Registry, Unilever Colworth Recruitment Database) and in addition for the English study to those who responded to adverts in newspapers. Subjects were selected to give an even spread of subjects across the chronological age range of each study and screened for eligibility criteria via a telephone interview.

For the Danish twin study, to be included, subjects were female, free of cuts, lacerations, abrasions, bruises, and excessive hair or blemishes on the face, had a surviving twin pair who was also willing to participate in the study, were in general good health, understood the test procedures and agreed to adhere to study requirements, were Caucasian, aged between 59 and 81 years, and were willing to participate in a study that involved non-invasive skin measurements and photography. The subjects were excluded if the subject had an existing skin condition such as psoriasis or eczema on the face, had participated in a study where the face was the target area in the previous 4 weeks, had ever had cosmetic or laser surgery on their face or hair transplant operations on their scalp, had used retinoic acid containing creams on their face or topical hair growth products on their scalp in the last 5 years, or were involved in any aspect of study administration (i.e. taking instrumental assessments).

For the British study, to be included subjects had to be female, between the ages of 45 and 75, Caucasian, in good general health, understand the test procedures and agree to adhere to study requirements, be free of cuts, lacerations, abrasions, bruises, and excessive hair or blemishes on the face, and be prepared to provide proof of age. Subjects were excluded if they had diabetes, were pregnant or breast feeding, had an active skin condition on the face or neck such as psoriasis or eczema, had tattoo make-up on the face, had a history of any type of skin cancer, were smokers, used botox, had ever had any surgery or laser treatment on the face or neck, had participated in a study where the face or neck was the target area within 4 weeks of the start of the study.

Before arriving at the study site, subjects were asked not to use any hair styling products or face products (e.g. creams, make-up) on or near the face or neck on the day of the study, self-tanning lotion on their face and neck within one week of the study visit, consume any hot or caffeinated beverages for one hour prior to the study visit, not to partake in vigorous exercise an hour before their study visit and to refrain from alcohol use 24 hours before the visit. Subjects were at rest for a minimum of 15 minutes before the photography and a facial wipe was used to remove excess sweat or any residual make-up from the face (also see [7]).

For the effects of hair colorants on perceived age, twins were dichotomized into those who reported hair colorant use (n=64) or those who reported no use (n=162), and the mean perceived age data for both groups compared by analysis of covariance with chronological age included as a covariate. For the hair graying measure, to maximize the number of twin pairs in the analyses, those who had reported not using hair colorants for a year before the study site visit (n=170) were included in the measurements. To measure the degree of hair graying in an image, the 45° photographic images of subjects were used to generate cropped images of hair from the temporal area of the left-side of the head (Fig. S2); five subjects were excluded at this point due to technical reasons. The color of the images was compared in red, green and blue (RGB) color space to images of known hair color (blonde, red, light brown, dark brown and black) and then allocated a color dependent on similarity. The pixels from each image were then classified as gray or non-gray dependent on whether they were more similar to gray hair or their previously allocated hair color. The classification was carried out in CIELAB color space using a linear discriminant classifier which incorporated specular light effects [51]. The percentage of pixels in the image that were identified as gray or specular gray was outputted. To ensure that the percentage gray allocated to each image were similar to human judgments, the results were quality checked by 2 assessors. Each assessor judged whether the image analysis was correct to within a 15% margin above and below their own judgments. Seven images failed both assessors’ checks and were excluded from subsequent analysis leaving a total of 158 subjects consisting of 62 intact twin pairs.

Sun-damage was graded according to a previously reported methodology for sun-damage [46]. Pigmented age spots were graded by a visual assessment of light, patchy, mottled hyper-pigmentation, actinic lentigines and solar freckling. The 9 point photographic scale was determined using quantitative and qualitative criteria such as the area/density of pigment, color intensity and uniformity of distribution. Skin wrinkling was graded on a 9-point scale by a visual assessment of the number and depth of fine and course wrinkles (i.e. shallow indentations or lines, deep lines, furrows or creases). Fine wrinkles tend to appear in periorbital and perioral regions and had a small weighting in the 9 point photographic scale; coarse wrinkles had a strong weighting in the scale and tend to appear on the forehead, glabella, chin, nasolabial and periorbital areas and are larger and closer to the eyes and mouth than are fine wrinkles.

**Technical Reproducibility**

Perceived age reproducibility for the methodology utilized here has been previously reported and has been demonstrated to be highly reproducible when large numbers of assessors are used [7]. Here, the Cronbach’s Alpha test demonstrated high reproducibility between individual assessors (main manuscript Table 3). Lip height was measured in 10 photographic images independently by two assessors and the scores compared. A high correlation was found between the scores (main manuscript Table 3) indicating good reproducibility for lip height measures from photographic images. There was also a high correlation between scores from 10 skin replicas analyzed at the start and again at the end of the skin replica PRIMOS measurement period (main manuscript Table 3). Inter-assessor agreement between dermatologists for grading of sun-damage has been shown to be fair [46]. Here, 10 twin images were present in the skin grading sequence twice (the dermatologist was blind to the design) and the duplicate images scored over 4 months apart. The intra-assessor reproducibility for these duplicate images was good for all three skin grade measures (main manuscript Table 3).

There have been no previous reports of inter-assessor agreements for hair recession or thinning on the vertex in women. The reproducibility scores reported here were fair (main manuscript Table 2) suggesting general agreements between the assessors, although the hair thinning scores were the least reproducible of any of the measures. As there have been no previously reported reproducibility or heritability estimates for these hair measures in women, further studies in other populations will be required to confirm the findings reported here.

To determine the accuracy of the hair graying model, three assessors independently scored hair graying for 15 subjects. Each subject’s hair was classified as either no gray, 25%, 50%, 75% or 100% gray, and the computer scores for these 15 subjects were similarly classified. These computer percentile scores were compared to the median of the human assessor percentile scores by Spearman correlation analysis and found to be very similar (main manuscript Table 3). Therefore, the hair graying image analysis employed here is equivalent to human assessments of hair graying.

50. Messenger AG, Sinclair R (2006) Follicular miniaturization in female pattern hair loss: clinicopathological correlations. Brit J Dermatol 155: 926-930.

51. Duda RO, Hart PE, and Stork DG (2001) Pattern Classification. Second edition, John Wiley and Sons, New York.
