# Supplementary figures and images for: Why Some Women Look Young for Their Age
Source: PLoS One. 2009 Dec 1;4(12):e8021. doi: 10.1371/journal.pone.0008021 (PMC2779449; doi:10.1371/journal.pone.0008021)

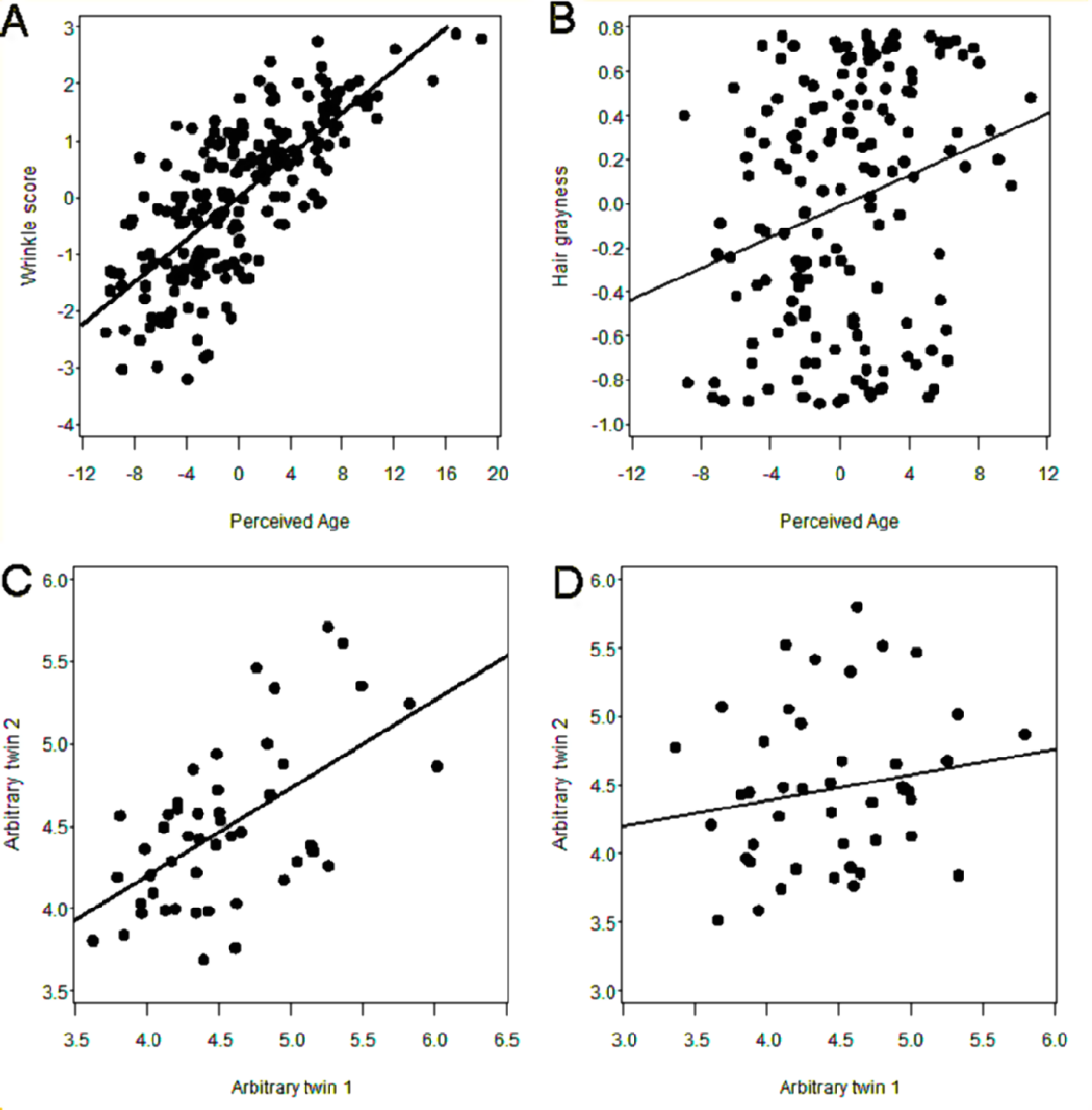

Supplement: Figure S1 — Scatter plots representative of correlations presented in the main manuscript. a) Facial wrinkling versus perceived age from facial images after adjusting for chronological age. b) Hair grayness versus perceived age from passport-type images after adjusting for chronological age. c) Wrinkle depth measures from beside the left eye for monozygotic twins (x-axis) and their sisters (y-axis). d) Wrinkle depth measures from beside the left eye for dizygotic twins (x-axis) and their sisters (y-axis). Graph lines are linear fits of the y-axis data onto the x-axis data and are representative of the correlation values in the main manuscript. (6.20 MB TIF) [file pone.0008021.s002.tif]

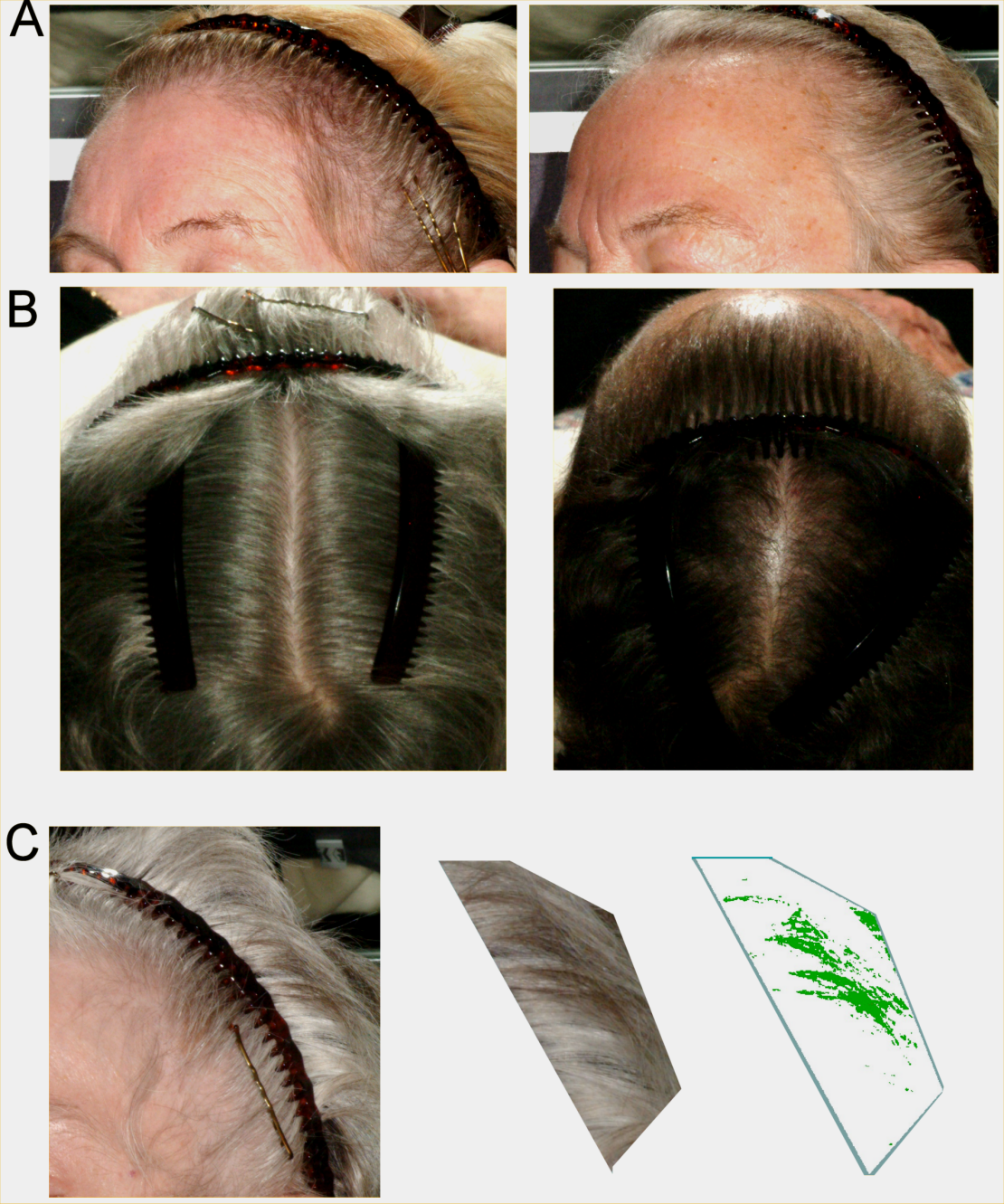

Supplement: Figure S2 — Representative images for hair aging measures. a) A 45-degree angle image of a subject with little to no hair recession (left-hand image) and a subject with grade 3 recession on the Hamilton-Norwood scale (right-hand side). b) An image of a subject with little to no hair thinning (left-hand image) and a subject with grade 3 thinning on the Sinclair scale (right-hand side). c) Example of a 45-degree image (left hand image) used to extract an area of hair from the left-hand temporal area of the head (centre image) and image analysis of gray (white) and non-gray (green) pixels in the image (right-hand image). (4.43 MB TIF) [file pone.0008021.s003.tif]
