# Supplementary material for: Why Some Women Look Young for Their Age
Source: PLoS One. 2009 Dec 1;4(12):e8021. doi: 10.1371/journal.pone.0008021 (PMC2779449; doi:10.1371/journal.pone.0008021)
Supplement: Table S1 — Aging appearance feature and chronological age unadjusted inter-correlation values. (0.05 MB DOC) [file pone.0008021.s004.doc]

**Table S1**: Aging appearance feature and chronological age unadjusted inter-correlation values.

| **Twin Study** |  |  |  | **British Study** |  |  |  |  |  |  |
| --- | --- | --- | --- | --- | --- | --- | --- | --- | --- | --- |
|  | Chronological Age |  |  |  |  |  |  |  |  |  |
| Pigmented spots | 0.06 | Pigmented spots |  | Pigmented spots | **0.49***** | **0.29***** | 0.08 | **-0.16*** | **0.18*** | 0.11 |
| Sun-damage | **0.14*** | **0.31***** | Sun-damage |  | Sun-damage | **0.90***** | **0.48***** | **-0.37***** | **0.72***** | **0.64***** |
| Wrinkles | **0.18**** | **0.16*** | **0.92***** | Wrinkles |  | Wrinkles | **0.50***** | **-0.42***** | **0.75***** | **0.66***** |
| Wrinkle depth | 0.05 | 0.00 | **0.43***** | **0.46***** | Wrinkle depth |  | Wrinkle depth | **-0.26**** | **0.53***** | **0.45***** |
| Lip height | **-0.34***** | 0.00 | -0.08 | -0.09 | -0.04 | Lip height |  | Lip height | **-0.45***** | **-0.31***** |
| Perceived age^†^ | **0.60***** | 0.12 | **0.63***** | **0.69***** | **0.33***** | **-0.33***** | Perceived age^†^ |  | Perceived age^†^ | **0.89***** |
| Perceived age^‡^ | **0.69***** | 0.05 | **0.29***** | **0.33***** | **0.16*** | **-0.31***** | **0.73***** | Perceived age^‡^ |  | Chronological age |
| Hair graying | 0.06 | 0.03 | -0.11 | -0.11 | -0.06 | -0.06 | -0.10 | **0.23**** | Hair graying |  |
| Hair recession | 0.17 | 0.11 | 0.02 | 0.05 | 0.06 | -0.06 | **0.20**** | **0.23**** | 0.04 | Hair recession |
| Hair thinning | -0.03 | 0.04 | 0.00 | 0.02 | 0.14 | -0.06 | -0.01 | 0.10 | **0.29***** | **0.20**** |

* <0.05, ** <0.01, *** <0.001, ^†^ – facial image derived, ^‡^ – passport-type image derived.
