# Supplementary material for: Why Some Women Look Young for Their Age
Source: PLoS One. 2009 Dec 1;4(12):e8021. doi: 10.1371/journal.pone.0008021 (PMC2779449; doi:10.1371/journal.pone.0008021)
Supplement: Table S2 — Multivariate linear modeling to predict perceived age excluding hair graying or facial wrinkle measures. (0.03 MB DOC) [file pone.0008021.s005.doc]

### Table S2. Multivariate linear modeling to predict perceived age excluding hair graying or facial wrinkle measures.

| **Feature** | **Perceived age Danish twins (n=204) ^†^** | **Perceived age Danish twins passport type images (n=158)^‡^** | **Perceived age British subjects (n=162)^†^** |
| --- | --- | --- | --- |
| Chronological age | 0.55 (0.05)*** | 0.55 (0.06)*** | 0.72 (0.04)*** |
| Pigmented spots | -0.49 (0.20)* | N/S | -0.47 (0.21)* |
| Sun-damage | 3.13 (0.23)*** | N/S | 2.05 (0.31)*** |
| Wrinkles | N/A | 0.83 (0.23)*** | N/A |
| Lip height | -0.52 (0.18)** | N/S | -0.91 (0.17)*** |
| Hair graying | N/S^§^ | N/A | N/A |
| Hair recession | 1.18 (0.42)** | N/S | N/A |
| Hair thinning | N/S | 1.09 (0.52)* | N/A |

*<0.05, **<0.01, ***<0.001, N/S - not significant in the model, N/A - not available, ^§^ - see Methods main manuscript section 3.4, ^†^ excluding facial wrinkles, ^‡^ excluding hair graying.
